# COMMUNICATING DEMENTIA ACROSS THREE GENERATIONS WITHIN CHINESE FAMILIES

**DOI:** 10.1093/geroni/igae098.4142

**Published:** 2024-12-31

**Authors:** Shicheng Xu, Vivian Weiqun Lou, Ernest Gonzales

**Affiliations:** New York University, New York, New York, United States; The University of Hong Kong, Hong Kong, China (People’s Republic); New York University, New York, New York, United States

## Abstract

Despite the benefit of early detection and diagnosis of dementia, globally, nearly 75% of people living with dementia are not diagnosed, indicating the barriers to help-seeking behaviors in the early stages. While in individualistic culture, help-seeking is subject to self-determination, in collectivist culture like China, help-seeking is subject to family determination that requires communication among multigenerational family members, including adult grandchildren. Research focusing on pre-diagnostic family communication is scarce. We aimed to understand how the family communicated to get the initial screening, disclose the diagnostic result, address the uncertainties, and manage emotions across three generations. Guided by constructivist grounded theory, we conducted semi-structured interviews with 28 Chinese participants (6 adult grandchildren, 5 adult children, 9 people with early-stage dementia, and 7 spouses). The results revealed “disconnected” and “connected” family communication patterns between the message sender and receiver. Barriers to connected communication include hierarchical family relationships, limited emotional expression, preference for implicit and crisis-induced communication. Family members tend to put an invisibility clock on dementia symptoms, the diagnostic result, and even the self-consciousness of people living with dementia. Nonetheless, older adults who received an early-stage dementia diagnosis expressed a strong desire to engage in family communication. Adult grandchildren also exerted a key role in family communication and decision-making processes. Social workers should empower intergenerational family members with communication and persuasion strategies to enhance diagnosis, prognosis, and emotional communication. This study underscores the fundamental role of family communication in early-stage dementia to increase screening and facilitate the subsequent care arrangement.

